# Prognostic Value of the Lung Immune Prognostic Index May Differ in Patients Treated With Immune Checkpoint Inhibitor Monotherapy or Combined With Chemotherapy for Non-small Cell Lung Cancer

**DOI:** 10.3389/fonc.2020.572853

**Published:** 2020-10-09

**Authors:** Wenxian Wang, Zhangzhou Huang, Zongyang Yu, Wu Zhuang, Weijun Zheng, Zhijian Cai, Lei Shi, Xinmin Yu, Guangyuan Lou, Wei Hong, Yiping Zhang, Ming Chen, Zhengbo Song

**Affiliations:** ^1^Department of Medical Oncology, Cancer Hospital of the University of Chinese Academy of Sciences & Zhejiang Cancer Hospital, Hangzhou, China; ^2^Institute of Cancer and Basic Medicine, Chinese Academy of Sciences, Hangzhou, China; ^3^Department of Medical Oncology, Fujian Provincial Cancer Hospital, Fujian Medical University Cancer Hospital, Fuzhou, China; ^4^Department of Medical Oncology, The 900th Hospital, Fuzhou, China; ^5^Department of Medical Statistics, College of Public Health, Zhejiang Chinese Medical School, Hangzhou, China; ^6^Institute of Immunology, Zhejiang University School of Medicine, Hangzhou, China; ^7^Hangzhou YITU Healthcare Technology Co., Ltd., Hangzhou, China; ^8^Zhejiang Key Laboratory of Radiation Oncology, Zhejiang Cancer Hospital, Hangzhou, China

**Keywords:** immune checkpoint inhibitor, non-small cell lung cancer, biomarker, chemotherapy, lung immune prognostic index

## Abstract

**Background:**

Lung immune prognostic index (LIPI) status was recently developed to predict responses to immune checkpoint inhibitor (ICI) treatments. However, it is unclear whether LIPI is a prognostic index for both patients treated with ICI monotherapy and patients treated with ICIs combined with chemotherapy (ICIs CC).

**Methods:**

This retrospective study established the patterns of LIPI in Chinese patients with advanced non-small cell lung cancer. Lung immune prognostic index based on the derived neutrophil-to-lymphocyte ratio greater than 3 and lactate dehydrogenase greater than the upper limit of normal was developed to characterize good, intermediate, or poor LIPI status. Associations between LIPI status and progression-free survival (PFS) and overall survival (OS) were analyzed. Kaplan–Meier curves and Cox proportional hazards models were used to determine survival differences.

**Results:**

Three hundred thirty patients were included in this study. Of these patients, 216 received ICI monotherapy and 114 received ICIs CC. A good LIPI status was associated with better PFS (6.1 months vs. 2.3 months vs. 2.1 months, *P* = 0.023) and OS (24.2 months vs. 14.5 months vs. 9.3 months, *P* < 0.001) in ICI monotherapy compared to intermediate or poor LIPI status. No differences in PFS (17.9 vs. 9.9 months vs. 7.6 months, *P* = 0.355, respectively) and OS (*P* = 0.346) were observed in patients who received ICIs CC. Moreover, we found that patients who had an improved LIPI status compared with the baseline value had a longer PFS with ICI monotherapy and LIPI intermediate status (8.4 months vs. 2.1 months vs. 1.4 months, *P* < 0.001). However, in patients treated with ICIs CC, these dynamic changes were not observed (*P* = 0.444).

**Conclusions:**

Lung immune prognostic index status and dynamic changes in LIPI could be prognostic markers of treatment response to ICI monotherapy, but not to ICIs CC. In particular, good LIPI status was associated with a better clinical outcome compared with intermediate and poor LIPI status in ICI monotherapy treatment.

## Introduction

Due to the rapid development of immunotherapy, significant improvements in efficacy and survival have been made in non-small cell lung cancer (NSCLC) patients treated with immune checkpoint inhibitors (ICIs) (1, 2). Previous research reported an objective response rate (ORR) ranging from 14 to 20% in NSCLC patients who were treated with single-agent ICIs as second-line therapy before programmed death-ligand 1 (PD-L1) biomarker selection, with a median overall survival (OS) ranging from 10 to 12 months (3–6). Nowadays, ICIs combined with chemotherapy is a more attractive choice for first-line therapy as the ORR following treatment is approximately 50% (7–9). It is known that PD-L1 expression is one of the most studied biomarkers and is recommended as a standard biomarker for ICIs (3, 10). Immunohistochemistry (IHC) is the most common strategy used to identify cells with PD-L1 expression and thereby predict response to immunotherapy. Five clones have mainly been used in various trials for PD-L1 estimation-22C3, 28-8, SP263, SP142, and 73-10. The first three assays showed concordance in a major comparative analysis (11). Of these, the Dako 22C3 pharmDx (Agilent Technologies/Dako, Carpinteria, CA, United States), the companion platform in pembrolizumab studies, has been most validated for the purpose (12). Nevertheless, several factors including tumor heterogeneity and PD-L1 negative patients also responding to ICIs are limiting its predictive value. Potentially, tumor mutational burden (TMB) may be an independent factor of PD-L1 expression in predicting ICIs efficacy (13). However, it has been shown that TMB may also have histologic and spatial heterogeneity, with squamous NSCLC having higher TMB than non-squamous NSCLC, metastatic sites having higher TMB than primary sites, and brain metastasis having higher TMB than other metastatic sites (14). Further studies are awaited to generate a consensus on how best to utilize TMB in clinical practice. Also, tumor-infiltrating lymphocytes (TILs) (15) and spatial TIL (SpaTIL) (16) are evolving predictive biomarkers of ICI response that warrant further evaluation. Therefore, biomarkers may have different prognostic efficacy in patients treated with ICIs. Furthermore, the detection of both TMB and PD-L1 status still has problems related to test acquisition, method standards, and testing time. It is also more difficult to obtain tumor specimens during dynamic detection. Therefore, a non-invasive marker that can dynamically and conveniently predict the efficacy of immunotherapy is required.

Cancer-associated inflammation leads to poor survival, and different inflammatory biomarkers in the blood such as neutrophils and lymphocytes have been studied as potential prognostic indicators in various cancers. The neutrophil-to-lymphocyte ratio (NLR) has been studied as a marker of systemic inflammation in the prognosis of various malignancies (17). Previous studies have indicated the importance of the NLR and baseline lactate dehydrogenase (LDH) level in determining the outcomes of patients with various cancers (18, 19). Mezquita et al. found that the derived NLR (dNLR) was associated with disease control and treatment response to ICIs in NSCLC. To strengthen the prognostic power of the dNLR in NSCLC (20), a lung immune prognostic index (LIPI) was developed recently based on 466 patients with a test (*n* = 161) and a validation set (*n* = 305) who received ICIs. The researchers found that the LIPI status was associated with survival in ICI-treated patients. Kazandjian and colleagues performed an exploratory retrospective analysis of the LIPI on pooled clinical trial data from studies evaluating ICIs or targeted therapy (TT) in NSCLC studies submitted to the FDA (21). They suggested that the baseline LIPI score may be a prognostic biomarker irrespective of treatment in patients with metastatic NSCLC. However, they did not assess changes in LIPI score over time with treatment. On the other hand, in previous studies, dynamic changes in the NLR during treatment were associated with survival (22, 23). Patients with a high post-treatment NLR had significantly shorter progression-free survival (PFS) than those with a low post-treatment NLR. Therefore, dynamic changes in the LIPI during treatment require further investigation.

Previous studies have focused on the correlation between immune monotherapy and various cancer and inflammation indicators. However, more and more patients are now receiving combination therapies. For example, the pembrolizumab–chemotherapy combination is the preferred therapy in the subgroup with PD-L1 1–49% expression and <1% for both non-squamous and squamous histology (24, 25). In addition, there are few reports on whether the dynamic changes in LIPI during ICI treatment can predict the efficacy of ICIs. Riedl et al. (26) reported LIPI and ICI treatment outcomes in 87 patients with advanced NSCLC who were treated with ICIs at a single academic center in Austria. This study externally validated an elevated LIPI as a biomarker of poor ICI treatment outcomes in patients with advanced NSCLC. The LIPI increases before disease progression. In this study, only the relationship between dynamic changes in LIPI and ICIs monotherapy was analyzed. In the present study, we performed a retrospective analysis to examine the prognostic value of the LIPI and dynamic changes in LIPI in NSCLC patients treated with ICI monotherapy and ICIs CC. This retrospective study was carried out to verify the associations between the LIPI status and clinical survival outcomes in Chinese patients with advanced NSCLC.

## Materials and Methods

### Patients

We conducted a retrospective study of a cohort of 330 patients with advanced NSCLC receiving treatment with programmed cell death protein 1 (PD-1)/PD-L1 inhibitors in a variety of settings, covering routine clinical care, expanded access, and compassionate-use programs, as well as clinical trials (nivolumab, pembrolizumab, atezolizumab, and other PD-1/PD-L1 inhibitors were used in clinical trials) between March 2016 and July 2019 in Zhejiang Cancer Hospital, Fujian Provincial Cancer Hospital and The 900th Hospital. This study was conducted in accordance with the Declaration of Helsinki. Patients who met the following inclusion criteria were included in the study: (1) Recorded clinicopathological information, including smoking history, age, gender, and histological type of lung cancer. (2) Pathologic examination of tumor specimens carried out with proven records of Epidermal Growth Factor Receptor (EGFR)/Anaplastic Lymphoma Kinase (ALK) gene status. (3) ICIs combined with platinum-based chemotherapy were first-line regimens. Patients were excluded if (1) clinical data including age, gender, and stage were missing; (2) pathologic examination showed small cell lung cancer; (3) results of gene detection showed EGFR mutation including EGFR 19 deletion, EGFR 21L858R mutation, or other uncommon EGFR mutations and ALK rearrangement; and (4) ICIs combined with non-platinum-based chemotherapy/antiangiogenic agents were as first-line regimens or ICIs combined with chemotherapy/antiangiogenic agents were as second-line regimens. The histologic classification of NSCLC was based on the World Health Organization criteria (2015 version) (27). Indicators, such as general characteristics, laboratory indicators, evaluable efficacy, and survival, were measured.

Complete blood cell counts, LDH, and albumin levels at baseline before ICI treatment (within 7 days of the first treatment) were extracted from electronic medical records. The LIPI was developed using the dNLR (greater than three) and LDH (exceeding the ULN), and the population was then divided into three risk groups according to the sum of these two factors: good (0 factor), intermediate (1 factor), and poor (2 factors) [good was dNLR > 3 and LDH < ULN; intermediate was dNLR ≤ 3 and LDH ≥ ULN or dNLR > 3 and LDH < ULN; poor was dNLR > 3 and LDH ≥ ULN (28)], and the threshold for LDH varied in each center according to their own ULN. We also obtained the dNLR and LDH values prior to patients receiving their second cycle of treatment to assess the dynamic changes in LIPI. A dynamic change in LIPI was defined as the difference in the baseline LIPI compared with the second LIPI score during the second cycle of treatment, and the changes were categorized as follows: better, stable, and worse. Programmed death-ligand 1 expression was analyzed in tumor cells by IHC, and an expression of at least 1% was considered positive. Programmed death-ligand 1 immunoassays were performed at each hospital using a monoclonal antibody against PD-L1 (22C3 PharmDx; Agilent Technologies, Santa Clara, CA, United States).

The authors are accountable for all aspects of the work in ensuring that questions related to the accuracy or integrity of any part of the work are appropriately investigated and resolved. Institutional review board approval was obtained independently at each center and was in accordance with the guidelines of the Helsinki Declaration (as revised in 2013), and individual consent for this retrospective analysis was waived.

### Treatments and Response Assessment

We collected data on the enrolled NSCLC patients during their disease process, including chemotherapy regimens and ICIs. We divided the study population into two groups, including the ICI monotherapy group and the ICIs CC group. In the ICIs CC group, chemotherapy regimens were based on pathological subtypes and different clinical trials, and included pemetrexed, albumin paclitaxel, gemcitabine, docetaxel, carboplatin, and cisplatin. All chemotherapy regimens were calculated according to the standard dose of the NCCN guidelines. Response to each treatment was assessed using Response Evaluation Criteria in Solid Tumors (RECIST v1.1). Before analysis, efficacy evaluation was examined by two oncologists who evaluated the tumor response according to RECIST 1.1.

### Statistical Analysis

Progression-free survival was defined as the period from the initial date of ICIs drug treatment to confirmation of disease progression or death. Overall survival was determined from the date of confirmed advanced NSCLC to death or last follow-up evaluation.

Baseline characteristics stratified by LIPI status were described as frequency and percentages (for categorical variables) and median and interquartile (for abnormally distributed continuous variables). Kaplan–Meier estimates and the log-rank test were used to evaluate PFS and OS. In addition, a series of Cox proportional hazards regression models was performed to examine which factors were independently associated with PFS and OS. All statistical analyses were performed using SPSS (version 25.0; SPSS, Inc., Chicago, IL, United States). Two-sided *P* values < 0.05 were considered statistically significant. The last follow-up date was January 30, 2020.

## Results

### Patient Characteristics

Three hundred thirty patients with advanced NSCLC were included in this study and 216 of these patients received ICI monotherapy, and 114 patients received ICIs CC. The chemotherapy regimens were all platinum-based chemotherapy.

In the ICI monotherapy group, the median baseline LDH value was 229 U/L and 94 patients (43.5%) had an LDH level greater than the ULN. In the ICIs CC group, the median baseline LDH value was 230 U/L and 49 patients (43.0%) had an LDH level greater than the ULN. Baseline neutrophil count was 4.3 × 10^9^/L and 5.1 × 10^9^/L, respectively. Fifty-three patients (24.5%) had a baseline dNLR greater than 3 in the ICI monotherapy group, and 29 patients (25.4%) had a baseline dNLR greater than 3 in the ICIs CC group. Further detailed baseline characteristics in the three LIPI groups are summarized in Table 1. With regard to PD-L1 expression, 108 patients (108/330, 32.7%) were tested and 66 patients (66/108, 61.1%) showed positive PD-L1 expression. In the ICI monotherapy group, 63.2% (43/68) of patients showed positive PD-L1 expression and 57.5% (23/40) of patients in the ICIs CC group showed positive PD-L1 expression.

**TABLE 1:** Baseline characteristics according to LIPI status in the ICI monotherapy and ICIs combined with chemotherapy groups.

	ICI monotherapy group (*n* = 216)				ICIs combination chemotherapy group (*n* = 114)			
	LIPI 0	LIPI 1	LIPI 2	*P* value	LIPI 0	LIPI 1	LIPI 2	*P* value
	Good	Intermediate	Poor		Good	Intermediate	Poor	
	(*n* = 98)	(*n* = 87)	(*n* = 31)		(*n* = 51)	(*n* = 48)	(*n* = 15)	
**Sex**				0.191				0.213
Male	77 (78.6%)	77 (88.5%)	25 (80.6%)		43 (84.3%)	42 (87.5%)	13 (86.7%)	
Female	21 (21.4%)	10 (11.5%)	6 (19.4%)		8 (15.7%)	6 (12.5%)	2 (13.3%)	
**Age, years**				0.597				0.735
<65	70 (71.4%)	57 (65.5%)	20 (64.5%)		36 (70.6%)	32 (66.7%)	9 (60.0%)	
ł65	28 (28.6%)	30 (34.5%)	11 (35.5%)		15 (29.4%)	16 (33.3%)	6 (40.0%)	
**Smoking history**				0.623				0.989
No	25 (25.5%)	20 (23.0%)	10 (32.3%)		7 (13.7%)	7 (14.6%)	2 (13.3%)	
Yes	73 (74.5%)	67 (77.0%)	21 (67.7%)		44 (86.3%)	41 (85.4%)	13 (86.7%)	
**ECOG score**				0.548				1
0–1	93 (94.9%)	79 (90.8%)	29 (93.1%)		51 (100.0%)	48 (100.0%)	15 (100.0%)	
2	5 (5.1%)	8 (9.2%)	2 (6.9%)		0	0	0	
**Histologic subtype**				0.913				0.943
Adenocarcinoma	45 (45.9%)	40 (46.0%)	16 (51.6%)		22 (43.1%)	20 (41.7%)	7 (46.7%)	
Squamous	49 (50.0%)	40 (46.0%)	12 (38.7%)		24 (47.1%)	26 (54.2%)	7 (46.7%)	
Not otherwise specified (NOS)	4 (4.1%)	7 (8.0%)	3 (9.7%)		5 (9.8%)	2 (4.2%)	1 (6.7%)	
**Lines of ICIs**				0.238				1
1	10 (10.2%)	11 (12.6%)	1 (3.2%)		51 (100.0%)	48 (100.0%)	15 (100.0%)	
2	78 (79.6%)	70 (80.5%)	26 (83.9%)		0	0	0	
ł3	10 (10.2%)	6 (6.9%)	4 (12.9%)		0	0	0	
**PD-L1 status**				0.471				0.267
Negative	12 (12.2%)	9 (10.3%)	5 (16.1%)		10 (19.6%)	6 (12.5%)	1 (6.7%)	
Positive	15 (15.3%)	24 (27.6%)	4 (12.9%)		11 (21.6%)	10 (20.8%)	2 (13.3%)	
Unknown	71 (72.4%)	54 (62.1%)	22 (71.0%)		30 (58.8%)	32 (66.7%)	12 (80.0%)	
**Stage at ICI treatment**				0.309				0.906
IIIB/IIIC	24 (24.5%)	14 (16.1%)	5 (16.1%)		19 (37.3%)	19 (39.6%)	5 (33.3%%)	
IV	75 (75.7%)	73 (83.9%)	26 (83.9%)		32 (62.7%)	29 (60.4%)	10 (66.7%)	
**Liver metastasis**				0.006				0.614
Yes	4 (4.3%)	17 (20.2%)	4 (13.3%)		6 (11.8%)	9 (18.8%)	2 (86.7%)	
No	88 (95.7%)	67 (79.8%)	26 (86.7%)		45 (88.2%)	39 (81.3%)	13 (86.7%)	
**Brain metastasis**				0.567				0.298
Yes	17 (18.9%)	14 (17.1%)	3 (10.3%)		6 (11.8%)	3 (6.3%)	3 (20.0%)	
No	73 (81.1%)	68 (82.9%)	26 (89.7%)		45 (88.2%)	45 (93.8%)	12 (80.0%)	

### Analysis of ICI Monotherapy Treatment

**FIGURE 1:**
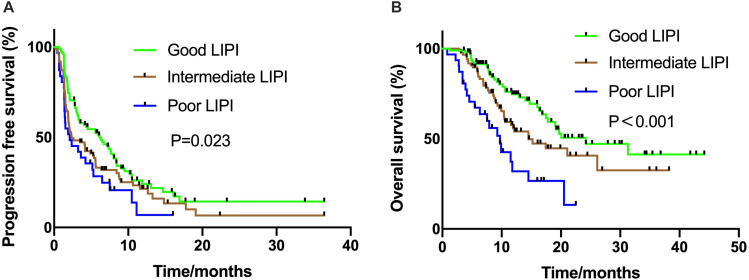
Kaplan Meier estimates of progression-free survival (PFS) and overall survival (OS) according to the Lung Immune Prognostic Index (LIPI) groups in patients with ICI monotherapy **(A)** PFS in the ICI monotherapy group (*P* = 0.023); **(B)** OS in the ICI monotherapy group (*P* < 0.001).

The median PFS was 4.10 (95% CI: 2.59–5.61) months. The LIPI was statistically related to PFS (*P* = 0.023) and the median PFS ranged from 2.1 months in the group with poor LIPI status to 6.1 months in the group with good LIPI status (Figure 1A). Similarly, associations between LIPI status and OS were observed (24.2 months vs. 14.5 months vs. 9.7 months, *P* < 0.001, respectively) (Figure 1B).

In the patients treated with ICIs monotherapy (*n* = 216), we analyzed sex, age, LIPI status, liver metastasis, brain metastasis, smoking status, the Eastern Cooperative Oncology Group (ECOG) performance status, histology, stage, PD-L1 status, and lines of ICI treatment in the univariate analysis of PFS. The results showed that LIPI status and ECOG performance score were independent prognostic factors. The details are shown in Table 2. Multivariate analyses indicated that ECOG score and poor LIPI status were independently associated with PFS, while smoking status, age, liver metastasis, and other factors were not independent prognostic factors in the ICI monotherapy group (Table 2).

**TABLE 2:** Univariable and multivariable analyses of progression-free survival (PFS) and overall survival (OS) in patients with ICIs monotherapy.

	Univariable model			Multivariable model		
	HR	95% CI	*P* value	HR	95% CI	*P* value
**PFS: Cox regression analysis (*N* = 216, 165 progression events)**						
Age	1.01	0.73–1.42	0.915	1.08	0.74–1.57	0.668
Sex	0.90	0.60–1.36	0.648	1.08	0.51–2.29	0.832
Smoking	0.86	0.61–1.23	0.427	0.88	0.47–1.64	0.704
Histology			0.249			0.281
Squamous (vs. Adenocarcinoma)	0.82	0.59–1.12	0.222	0.79	0.53–1.17	0.251
Other (vs. Adenocarcinoma)	0.61	0.30–1.22	0.166	0.57	0.26–1.23	0.154
ECOG score	1.84	1.03–3.28	0.037	1.92	1.02–3.62	0.043
Stage (III/IV)	1.15	0.78–1.69	0.475	1.09	0.67–1.78	0.702
LIPI status			0.023			0.098
Intermediate (vs. good)	1.36	0.97–1.9	0.069	1.24	0.85–1.80	0.254
Poor (vs. good)	1.80	1.14–2.83	0.011	1.70	1.04–2.79	0.033
Liver metastasis	1.58	0.98–2.53	0.056	1.58	0.94–2.65	0.080
Brain metastasis	1.18	0.78–1.78	0.420	1.06	0.68–1.66	0.775
PD-L1 status			0.741			0.707
Positive (vs. negative)	1.24	0.70–2.21	0.449	1.30	0.69–2.45	0.407
Unknown (vs. negative)	1.12	0.68–1.84	0.647	1.19	0.70–2.04	0.511
Lines of ICIs			0.280			0.646
First line (vs. ≥ three line)	0.60	0.29–1.23	0.184	0.69	0.31–1.51	0.355
Second line (vs. ≥ three line)	0.92	0.29–1.61	0.791	0.85	0.47–1.53	0.592

	**Univariable model**			**Multivariable model**		
	**HR**	**95% CI**	***P* value**	**HR**	**95% CI**	***P* value**

**OS: Cox regression analysis (*N* = 216, 95 death events)**						
Age	0.90	0.57–1.41	0.655	0.97	0.59–1.57	0.900
Sex	0.95	0.55–1.63	0.863	1.32	0.52–3.31	0.549
Smoking	0.85	0.54–1.35	0.510	0.82	0.39–1.69	0.592
Histology			0.045			0.142
Squamous (vs. Adenocarcinoma)	0.58	0.38–0.90	0.014	0.60	0.36–1.01	0.058
Other (vs. Adenocarcinoma)	0.66	0.28–1.55	0.346	0.56	0.20–1.51	0.254
ECOG score	1.64	0.79–3.41	0.184	1.28	0.55–2.95	0.559
Stage (III/IV)	1.14	0.66–1.99	0.624	1.07	0.54–2.11	0.831
LIPI status			< 0.001			< 0.001
Intermediate (vs. good)	1.58	1.00–2.49	0.048	1.67	0.99–2.81	0.052
Poor (vs. good)	3.29	1.88–5.75	< 0.001	3.54	1.88–6.66	< 0.001
Liver metastasis	1.08	0.60–1.95	0.779	0.79	0.40–1.52	0.482
Brain metastasis	1.32	0.79–2.20	0.276	1.17	0.65–2.11	0.582
PD-L1 status			0.703			0.656
Positive (vs. negative)	1.16	0.49–2.75	0.729	1.32	0.51–3.44	0.558
Unknown (vs. negative)	1.32	0.63–2.75	0.449	1.45	0.64–3.25	0.365
Lines of ICIs			0.114			0.136
First line (vs. ≥three line)	0.40	0.15–1.03	0.058	0.46	0.17–1.23	0.125
Second line (vs. ≥three line)	0.53	0.27–1.03	0.062	0.49	0.24–1.01	0.054

Univariate analysis demonstrated that LIPI status and histology were independent prognostic factors of OS and multivariate analyses showed that only LIPI status was a significant prognostic factor (Table 2).

### Analysis of ICIs Combined With Chemotherapy Treatment

**FIGURE 2:**
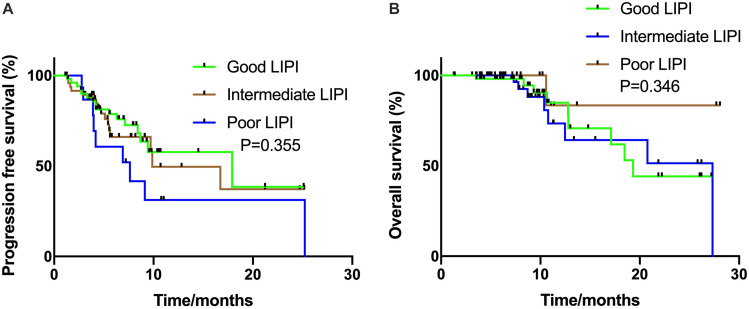
Kaplan Meier estimates of progression-free survival (PFS) and overall survival (OS) according to the Lung Immune Prognostic Index (LIPI) groups in patients with ICIs combination group **(A)** PFS in the ICIs combination group (*P* = 0.355); **(B)** OS in the ICIs combination group (*P* = 0.346).

In the ICIs CC group, all patients had an ECOG PS score of 0–1 and received ICIs combined with platinum-based chemotherapy, and the median PFS was 9.9 months. Unlike the ICI monotherapy group, the association between LIPI status and PFS was not statistically significant (17.9 vs. 9.9 months vs. 7.6 months, *P* = 0.355, respectively) (Figure 2A). The relationship between LIPI status and OS was not statistically significant (*P* = 0.346) (Figure 2B).

In the univariate analysis for PFS, we analyzed sex, age, LIPI status, liver metastasis, brain metastasis, smoking status, histology, stage, and PD-L1 status. The results showed that liver metastasis and brain metastasis were independent prognostic factors. Multivariate analyses indicated that liver metastasis and brain metastasis were also independently associated with PFS. The details are shown in Table 3.

**TABLE 3:** Univariable and multivariable analyses of progression-free survival (PFS) and overall survival (OS) in patients with ICIs combination chemotherapy.

	Univariable model			Multivariable model		
	HR	95% CI	*P* value	HR	95% CI	*P* value
**PFS: Cox regression analysis (*N* = 114, 41 progression events)**						
Age	1.63	0.87–3.06	0.124	1.82	0.94–3.54	0.075
Sex	1.83	0.70–4.78	0.211	0.49	0.01–12.42	0.667
Smoking	1.78	0.68–4.65	0.233	2.24	0.08–58.2	0.627
Histology			0.154			0.496
Squamous (vs. Adenocarcinoma)	1.87	0.96–3.64	0.063	1.63	0.72–3.65	0.236
Other (vs. Adenocarcinoma)	1.01	0.23–4.49	0.983	1.31	1.31–0.27	0.731
Stage (III/IV)	1.01	0.51–2.00	0.972	0.61	0.26–1.43	0.257
LIPI status			0.355			0.867
Intermediate (vs. good)	1.14	0.57–2.29	0.700	1.22	0.57–2.59	0.607
Poor (vs. good)	1.81	0.77–4.23	0.171	1.18	0.45–3.08	0.723
Liver metastasis	3.06	1.49–6.24	0.002	3.48	1.48–8.17	0.004
Brain metastasis	2.58	1.07–6.23	0.035	3.54	1.27–9.91	0.016
PD-L1 status			0.133			0.175
Positive (vs. negative)	0.30	0.07–1.22	0.093	0.37	0.08–1.62	0.189
Unknown (vs. negative)	1.02	0.42–2.49	0.951	1.21		0.706

	**Univariable model**			**Multivariable model**		
	**HR**	**95% CI**	***P* value**	**HR**	**95% CI**	***P* value**

**OS: Cox regression analysis (*N* = 114, 18 death events)**						
Age	0.81	0.30–2.18	0.679	0.50	0.16–1.58	0.242
Sex	1.22	0.34–4.31	0.752	0.00	0.00–2.93	0.962
Smoking	1.18	0.33–4.18	0.789	0.00	0.00–3.48	0.963
Histology			0.879			0.860
Squamous (vs. Adenocarcinoma)	1.27	0.50–3.24	0.612	1.41	0.40–4.91	0.584
Other (vs. Adenocarcinoma)	0.00	0.00–NR	0.983	0.00	0.00–2.81	0.970
Stage (III/IV)	0.68	0.23–1.94	0.474	0.51	0.14–1.76	0.289
LIPI status			0.346			0.346
Intermediate vs. good)	1.08	0.41–2.87	0.864	1.20	0.41–3.51	0.729
(Poor (vs. good)	0.24	0.02–2.16	0.206	0.20	0.00–2.14	0.186
Liver metastasis	2.94	1.02–8.40	0.044	4.33	1.05–17.75	0.042
Brain metastasis	1.01	0.22–4.56	0.981	3.08	0.47–20.19	0.241
PD-L1 status			0.723			0.997
Positive (vs. negative)	0.00	0.00–2.94	0.958	0.00	0.00–5.68	0.938
Unknown (vs. negative)	0.59	0.17–NR	0.421	0.99	0.22–4.38	0.999

*NR: not reached.*

Univariate analysis demonstrated that liver metastasis was an independent prognostic factor of OS and multivariate analyses showed that only liver metastasis was a significant prognostic factor (Table 3).

### Analysis of Dynamic LIPI Status in ICI Monotherapy and ICIs With Chemotherapy

In the ICI monotherapy group, 179 patients had dynamic LIPI status and we further analyzed the relationship between dynamic LIPI and PFS. The results are shown in Table 4. There was no statistically significant difference in the baseline good LIPI group and poor LIPI group. However, in the baseline intermediate LIPI group, PFS was 8.4, 2.1, and 1.4 months (*P* < 0.001), when C2 LIPI was good, stable, and poor, respectively. This dynamic change showed that the changes in PFS and LIPI were meaningful.

**TABLE 4:** The relationship between dynamic LIPI and PFS, and stratified LIPI before the second cycle of treatment (C2 LIPI).

C2 LIPI	ICI monotherapy			ICIs combination group		
	Baseline LIPI			Baseline LIPI		
	Good	Intermediate	Poor	Good	Intermediate	Poor
	(*n* = 77)	(*n* = 76)	(*n* = 26)	(*n* = 42)	(*n* = 43)	(*n* = 14)
Better	–	PFS = 8.4 months	PFS = 1.4 months	–	PFS = 9.7 months (*n* = 17)	PFS = 6.9 months
		(*n* = 15)	(*n* = 10)			(*n* = 7)
Stable	PFS = 9.0 months	PFS = 2.1 months	PFS = 2.1 months	PFS = 17.9 months	Not reached	PFS = 7.6 months
	(*n* = 54)	(*n* = 49)	(*n* = 16)	(*n* = 24)	(*n* = 14)	(*n* = 7)
Worse	PFS = 5.5 months	PFS = 1.4 months	–	PFS = 9.4 months	Not reached	–
	(*n* = 23)	(*n* = 12)		(*n* = 18)	(*n* = 12)	
*P* value	0.359	<0.001	0.997	0.808	0.444	0.773

To examine whether dynamic LIPI contributes to the prediction of OS or PFS after accounting for known prognostic factors in the ICI monotherapy group, we also fitted multivariable models including dynamic LIPI, histology, smoking, ECOG PS, liver metastasis, brain metastasis, and gender. For PFS, univariate and multivariate analyses showed that liver metastasis was an independent prognostic factor. For OS, lines of ICI treatment (*P* = 0.024) and dynamic LIPI (*P*<0.001) were independent prognostic factors (Supplementary Table 1).

We also analyzed the relationship between dynamic LIPI and PFS in the ICIs CC group. No statistical difference in PFS was observed for baseline LIPI to C2 LIPI (Table 4). Further univariate and multivariate analyses indicated that dynamic LIPI status was not an independent prognostic factor in the ICIs CC group. Liver metastasis was an independent prognostic factor (Supplementary Table 2). The univariate and multivariate analysis results of OS showed that dynamic LIPI status, histology, smoking, liver metastasis, brain metastasis, and gender were not independent prognostic factors.

## Discussion

In this study of NSCLC patients treated with ICI monotherapy or ICIs CC, we found that LIPI status was significantly associated with PFS and OS in the ICI monotherapy group, but not in the ICIs CC group. Our analysis suggests that the prognostic value of baseline LIPI status and its dynamic changes may be different between ICI monotherapy and ICIs CC in Chinese patients. Therefore, our study is the first report to evaluate baseline LIPI status and its dynamic changes between ICI monotherapy and ICIs CC in Asian patients.

We summarized published articles (18–23, 29–32) on the correlation between inflammation indicators and the efficacy of immunotherapy in patients with NSCLC (Supplementary Table 3). It can be seen that most of the studies focused on the relationship between LDH and LIPI status before treatment and the efficacy of immunotherapy, and mainly analyzed the correlation in patients receiving immuno-monotherapy. It is well-known that inflammation plays a critical role in cancer growth. In addition, LDH has been found to be associated with shorter survival (33–36). Mezquita et al. (20) reported that high LDH levels were associated with shorter OS in NSCLC patients treated with ICIs. In addition, a recent report (37) demonstrated that neutrophils were responsible for treatment failure following ICI therapy. Russo and colleagues (38) reported that a high dNLR was associated with no response to nivolumab, but not to docetaxel in NSCLC. According to previous reports, LIPI has a certain guiding significance in immunotherapy, but this may be different in chemotherapy. A recent study reviewed the clinical evidence supporting the use of the LIPI index as a clinically valuable biomarker for patients with NSCLC and other solid tumor types, treated with checkpoint inhibitors (39). Sorich et al. (23) demonstrated that pretreatment LIPI is a convenient prognostic marker able to identify atezolizumab-treated patient groups with significantly different survival and response outcomes. Another recent study explored the prognostic value of the LIPI in treatment-naive advanced NSCLC patients with high PD-L1 expression (≥50%) treated with pembrolizumab (40). Rubio et al. showed that the LIPI was able to define a group of patients with poor benefit from pembrolizumab monotherapy (40). However, LIPI is also a prognostic marker of survival and response in patients treated with chemotherapy; thus, it is not specifically prognostic for ICI treatment. Our study was designed to further identify whether this indicator had the same significance in ICI monotherapy and ICIs CC. The results showed that a poor LIPI status was associated with shorter PFS in NSCLC patients treated with ICI monotherapy. However, no such association was observed in the ICIs CC group. Therefore, the prognostic value of pretreatment LIPI status in monotherapy is relatively clear, and many studies have shown similar results. The prognostic value of LIPI status in immune-combined chemotherapy treatment is still unclear, and there are few related research reports. An abstract by Blanc-Durand et al. (41) reported in the 2019 ESMO conference indicated an association between LIPI and survival following first-line ICI single agent or in combination with chemotherapy in untreated advanced NSCLC patients. In the combination cohort (*n* = 98), 71 (72%) were male, with a median age of 66, and 84 (86%) had a PS ≤ 1. Based on LIPI (available for 69): 23 (33%) were in the good group, 34 (49%) were in the intermediate group, and 12 (17%) were in the poor group. In the chemotherapy combined with immunotherapy group, the sample size was not large with a total of 114 cases. Similar to our study, the population distribution was 51 (44.7%) in the good group, 48 (42.1%) in the intermediate group, and 15 (13.2%) in the poor group. Their results demonstrated that pretreatment LIPI correlates with survival in ICI combined with chemotherapy in advanced NSCLC patients (25.7 months vs 16.9 months vs 6.2 months, *P* = 0.02). The results of our OS analysis showed that the relationship between LIPI status and OS was not statistically significant (19.3 months vs 27.3 months vs NR, *P* = 0.393). Our results contradicted their study results even though both were retrospective articles, with unavoidable limitations. In addition, because there is no full text of their study, there were no general characteristics including PD-L1 expression, brain metastasis, and liver metastasis, as well as single factor and multivariate analysis. They also did not have results of a PFS analysis. Therefore, follow-up research is warranted to further examine the value of the LIPI in chemotherapy combined with immunotherapy treatment.

It is possible that the mechanism of combination therapy may be more complicated. Chemotherapy can modulate tumor immunity in a drug-dependent manner, suggesting that ICI chemotherapeutic regimens might influence the efficacy of immunotherapy, and different chemotherapy regimens might also influence the therapeutic outcome of immunotherapy. Moreover, considering the varied pharmacologic nature of chemotherapy drugs, it is possible that they also interfere with the anti-tumor immune process in different ways and may eventually lead to diverse consequences (42). Rassy et al. showed the schedule and sequence of chemotherapy, and ICI impacts tumor immunity. In fact, the rationale for the combination of chemotherapy and immunotherapy is poorly founded as chemotherapy is mostly cytotoxic and poorly induces mutations (43). Therefore, a single marker may not be enough to predict the treatment outcome of chemo-immunotherapy. Mezquita et al. (20) reported that pretreatment LIPI was correlated with a worse outcome following ICIs, but not chemotherapy. However, another report demonstrated that LIPI was also a prognostic marker of survival and response in patients treated with chemotherapy (44). A recent study demonstrated that LIPI was an independent prognostic factor of chemotherapy in lung adenocarcinoma (23). Therefore, the prognostic value of LIPI in chemotherapy is still controversial. Our results showed that the difference between the poor and good LIPI groups was greater in the ICI cohort than in the chemotherapy with ICI cohort, suggesting that immunologic status is more important in the prognostic assessment of ICI treatment. However, for chemotherapy combined with immunotherapy, prognostic assessment is more complex.

On the other hand, we analyzed the prognostic value of baseline LIPI and dynamic changes in LIPI before the second cycle of ICI or ICIs CC treatment. A study reviewed 54 NSCLC patients treated with anti-PD-1 antibodies and found that changes in the NLR after initiation of treatment were significantly correlated with PFS. Patients with a reduced NLR after anti-PD-1 antibody therapy had a higher ORR (43.2 vs. 22.2%) and longer PFS (6.2 vs. 3.0 months) (14). Another study examined the NLR of 88 NSCLC patients receiving ICIs and showed that patients who had a lower NLR at 8 weeks than at baseline were most likely to have a response (34). Mezquita et al. demonstrated that the early change in dNLR was correlated with outcomes in advanced NSCLC patients treated with immunotherapy (45). They demonstrated that dNLR at baseline, at cycle 2, and the change between these two time points was associated with outcomes in patients treated with immunotherapy independent of PD-L1 but not in patients treated with chemotherapy alone. dNLR is specifically prognostic in the context of immunotherapy. Riedl et al. (26) reported the LIPI and ICI treatment outcomes in 87 patients with advanced NSCLC who were treated with ICI monotherapy. This study validated an elevated LIPI as a biomarker of poor ICI treatment outcomes and the LIPI increased before disease progression. However, no study has investigated the significance of dynamic changes in LIPI with ICIs CC. We wondered if these dynamic changes could further identify the efficacy of ICI therapy. Interestingly, we found that the LIPI before the second cycle of treatment was better than at baseline, and patients had longer PFS in the group with baseline intermediate LIPI. However, following ICIs CC treatment, these dynamic changes were not correlated with efficacy. For ICIs in combination with chemotherapy, other markers need to be identified or a combination of multiple biomarkers should be evaluated. Our data showed that post-treatment LIPI was associated with improved PFS and OS in patients with NSCLC treated with ICI monotherapy in the group in which baseline LIPI was intermediate, but not with combination treatment. Thus, the sample size is also worth expanding or prospective research is worth carrying out to further confirm these findings.

To the best of our knowledge, this is the first study to evaluate the prognostic role of post-treatment LIPI in NSCLC, and our findings suggest that LIPI could be a potential predictor of response to ICI monotherapy, but not chemo-immunotherapy. However, the limitations of our study must be noted. The retrospective nature of this study may have influenced some results, including PD-L1 expression and the inclusion of heterogeneous patients, especially as only 32.7% of patients underwent analysis of tumor PD-L1 expression. It should be noted that most of the patients in the ICI monotherapy cohort were treated in the second- or third-line setting, in which PD-L1 status is not mandatory for prescribing the therapy. Furthermore, for patients with PD-L1 status, as this was not centrally performed, PD-L1 testing methodology was heterogeneous such as antibody measurements. In the ICIs CC cohort, most of the patients who had not arrived at the event may have affected the results of the statistical analysis, and we also await the results of our continued follow-up analysis. Therefore, this issue needs to be investigated further and the relationship between LIPI, other bio-markers such as TMB, and survival should be examined in the future.

## Conclusion

In summary, baseline LIPI status may be different in predicting efficacy in NSCLC patients treated with ICI monotherapy and ICIs combined with chemotherapy. Lung immune prognostic index status and its dynamic changes are significant prognostic biomarkers of the efficacy of single agent immunotherapy in patients with advanced NSCLC. However, LIPI status did not predict response outcomes in patients treated with chemo-immunotherapy. Further studies are needed to evaluate the prognostic role of LIPI in this setting.

## Data Availability Statement

The original contributions presented in the study are included in the article/Supplementary Material, further inquiries can be directed to the corresponding author/s.

## Ethics Statement

The studies involving human participants were reviewed and approved by the Institutional Ethics Committee at Zhejiang Cancer Hospital and each investigation site. Written informed consent from the participants’ legal guardian/next of kin was not required to participate in this study in accordance with the national legislation and the institutional requirements.

## Author Contributions

All authors listed have made a substantial, direct and intellectual contribution to the work, and approved it for publication. SZB, WWX and CM contributed to the study design. WWX, ZW, CZJ, SL, YXM, LGY, HW, ZYP, CM and SZB were responsible for interpretation of the results. HZZ, YZY, ZWJ and WWX contributed to statistical analysis. WWX, SZB, and ZWJ were prepared for the manuscript. All authors contributed to data collection and analysis.

## Conflict of Interest

LS was employed by the company Hangzhou YITU Healthcare Technology Co., Ltd. The remaining authors declare that the research was conducted in the absence of any commercial or financial relationships that could be construed as a potential conflict of interest.
