# Supplementary material for: Prognostic Value of the Lung Immune Prognostic Index May Differ in Patients Treated With Immune Checkpoint Inhibitor Monotherapy or Combined With Chemotherapy for Non-small Cell Lung Cancer
Source: Front Oncol. 2020 Oct 9;10:572853. doi: 10.3389/fonc.2020.572853 (PMC7583465; doi:10.3389/fonc.2020.572853)
Supplement: Supplementary file 1 [file Data_Sheet_1.docx]

**Supplement Table 1.** Univariable and multivariable analyses of progression free survival (PFS) and overall survival (OS) in patients with ICIs monotherapy.

| PFS: Cox regression analysis (N=179, 134 progression events) | | | | | | | | | |
| --- | --- | --- | --- | --- | --- | --- | --- | --- | --- |
|  |  | Univariable model | | |  | Multivariable model | | | |
|  |  | HR | 95%CI | P value |  | HR | 95%CI | P value | |
| Age | | 0.96 | 0.66-1.41 | 0.856 |  | 1.01 | 0.66-1.56 | | 0.931 |
| Sex | | 0.77 | 0.50-1.19 | 0.248 |  | 0.77 | 0.31-1.89 | | 0.576 |
| Smoking | | 0.81 | 0.57-1.20 | 0.312 |  | 1.01 | 0.48-2.13 | | 0.967 |
| Histology  Squamous (vs. Adencarcinoma)  other (vs. Adencarcinoma) | | 0.79  0.63 | 0.55-1.12  0.31-1.29 | 0.279  0.198  0.212 |  | 0.83  0.65 | 0.51-1.34  0.29-1.47 | | 0.558  0.455  0.309 |
| ECOG score | | 1.98 | 1.03-3.81 | 0.040 |  | 1.91 | 0.90-4.01 | | 0.088 |
| Stage (III/IV) | | 1.13 | 0.75-1.69 | 0.553 |  | 0.99 | 0.58-1.67 | | 0.991 |
| Dynamic LIPI variation  Stable  (vs. better)  Worse  (vs. better) | | 1.38  2.55 | 0.94-2.03  1.57-4.15 | 0.001  0.097  ＜0.001 |  | 1.29  2.54 | 0.84-1.97  1.47-4.38 | | 0.003  0.230  0.001 |
| Liver metastasis | | 1.82 | 1.10-3.00 | 0.019 |  | 2.07 | 1.18-3.64 | | 0.010 |
| Brain metastasis | | 1.10 | 0.69-1.78 | 0.687 |  | 1.00 | 0.59-1.69 | | 0.989 |
| PD-L1 status  Positive  (vs. negative)  Unknown  (vs. negative) | | 1.19  1.04 | 0.67-2.13  0.62-1.73 | 0.763  0.542  0.875 |  | 1.14  1.03 | 0.58-2.20  0.59-1.79 | | 0.901  0.696  0.913 |
| Lines of ICIs  First line  (vs. ≥three line)  Second line  (vs. ≥three line) | | 1.52  1.74 | 0.85-2.72  0.81-3.72 | 0.292  0.150  0.151 |  | 1.17  1.59 | 0.62-2.20  0.68-3.54 | | 0.545  0.621  0.289 |

| OS: Cox regression analysis (N=179, 71 death events) | | | | | | | | | |
| --- | --- | --- | --- | --- | --- | --- | --- | --- | --- |
|  |  | Univriable model | | |  | Multivariable model | | | |
|  |  | HR | 95%CI | P value |  | HR | 95%CI | P value | |
| Age | | 0.79 | 0.45-1.38 | 0.422 |  | 0.84 | 0.46-1.55 | | 0.590 |
| Sex | | 0.74 | 0.41-1.34 | 0.332 |  | 1.08 | 0.34-3.44 | | 0.888 |
| Smoking | | 0.83 | 0.49-1.42 | 0.512 |  | 1.07 | 0.40-2.85 | | 0.892 |
| Histology  Squamous (vs. Adencarcinoma)  other (vs. Adencarcinoma) | | 0.53  0.66 | 0.32-0.87  0.28-1.58 | 0.042  0.013  0.360 |  | 0.49  0.66 | 0.26-0.94  0.24-1.82 | | 0.105  0.034  0.428 |
| ECOG score | | 1.57 | 0.62-3.92 | 0.334 |  | 0.69 | 0.21-2.20 | | 0.538 |
| Stage (III/IV) | | 1.23 | 0.67-2.26 | 0.485 |  | 1.09 | 0.51-2.35 | | 0.807 |
| Dynamic LIPI variation  Stable  (vs. better)  Worse  (vs. better) | | 2.39  3.88 | 1.34-4.26  1.96-7.67 | ＜0.001  0.003  ＜0.001 |  | 3.27  6.01 | 1.66-6.45  2.66-13.54 | | ＜0.001  0.001  ＜0.001 |
| Liver metastasis | | 1.08 | 0.55-2.12 | 0.809 |  | 0.63 | 0.29-1.36 | | 0.243 |
| Brain metastasis | | 1.09 | 0.57-2.09 | 0.785 |  | 0.99 | 0.46-2.15 | | 0.991 |
| PD-L1 status  Positive  (vs. negative)  Unknown  (vs. negative) | | 1.08  1.27 | 0.45-2.60  0.60-2.70 | 0.745  0.847  0.520 |  | 0.96  1.26 | 0.36-2.56  0.55-2.88 | | 0.674  0.940  0.576 |
| Lines of ICIs  First line  (vs. ≥three line)  Second line  (vs. ≥three line) | | 1.56  2.89 | 0.67-3.64  1.04-8.02 | 0.094  0.297  0.041 |  | 1.31  3.36 | 0.50-3.44  1.18-10.15 | | 0.024  0.576  0.031 |

**Supplement Table 2.** Univariable and multivariable analyses of progression free survival (PFS) and overall survival (OS) in patients with ICIs combination chemotherapy.

| PFS: Cox regression analysis (N=99, 39 progression events) | | | | | | | | | |
| --- | --- | --- | --- | --- | --- | --- | --- | --- | --- |
|  |  | Univariable model | | |  | Multivariable model | | | |
|  |  | HR | 95%CI | P value |  | HR | 95%CI | P value | |
| Age | | 1.76 | 0.92-3.37 | 0.084 |  | 1.72 | 0.88-3.36 | | 0.111 |
| Sex | | 1.71 | 0.65-4.48 | 0.273 |  | 0.69 | 0.02-18.50 | | 0.830 |
| Smoking | | 1.65 | 0.63-4.33 | 0.304 |  | 1.67 | 0.06-44.33 | | 0.757 |
| Histology  Squamous (vs. Adencarcinoma)  other (vs. Adencarcinoma) | | 1.58  0.87 | 0.79-3.14  0.19-3.91 | 0.352  0.187  0.864 |  | 1.43  1.05 | 0.59-3.44  0.21-5.25 | | 0.715  0.416  0.947 |
| Stage (III/IV) | | 0.97 | 0.48-1.94 | 0.93 |  | 0.66 | 0.27-1.58 | | 0.354 |
| Dynamic LIPI variation  Stable  (vs. better)  Worse  (vs. better) | | 0.71  0.76 | 0.33-1.49  0.32-1.83 | 0.656  0.366  0.552 |  | 0.82  0.87 | 0.36-1.85  0.32-2.33 | | 0.894  0.639  0.787 |
| Liver metastasis | | 2.66 | 1.27-5.60 | 0.010 |  | 3.32 | 1.37-8.03 | | 0.007 |
| Brain metastasis | | 1.73 | 0.60-4.92 | 0.304 |  | 2.22 | 0.69-7.07 | | 0.176 |
| PD-L1 status  Positive  (vs. negative)  Unknown  (vs. negative) | | 0.38  0.95 | 0.09-1.60  0.36-2.49 | 0.313  0.189  0.919 |  | 0.86  0.37 | 0.29-2.55  0.11-1.28 | | 0.297  0.797  0.120 |

| OS: Cox regression analysis (N=99, 16 death events) | | | | | | | | | | |
| --- | --- | --- | --- | --- | --- | --- | --- | --- | --- | --- |
|  |  | Univriable model | | | |  | Multivariable model | | | |
|  |  | HR | | 95%CI | P value |  | HR | 95%CI | P value | |
| Age | | 0.74 | | 0.25-2.16 | 0.585 |  | 0.50 | 0.14-1.76 | | 0.286 |
| Sex | | 1.08 | | 0.30-3.88 | 0.902 |  | 0.94 | 0.00-2.10 | | 1.000 |
| Smoking | | 1.05 | | 0.29-3.78 | 0.939 |  | 1.63 | 0.00-3.66 | | 0.997 |
| Histology  Squamous (vs. Adencarcinoma)  other (vs. Adencarcinoma) | | 1.26  0.00 | | 0.46-3.41  0.00-NR | 0.900  0.656  0.985 |  | 0.92  0.00 | 0.17-4.86  0.00-8.78 | | 0.990  0.927  0.915 |
| Stage (III/IV) | | 0.54 | | 0.18-1.59 | 0.269 |  | 0.45 | 0.13-1.56 | | 0.214 |
| Dynamic LIPI variation  Stable  (vs. better)  Worse  (vs. better) | | 0.86  0.90 | | 0.18-3.94  0.23-3.45 | 0.981  0.846  0.885 |  | 1.03  0.67 | 0.24-4.29  0.07-6.52 | | 0.921  0.965  0.743 |
| Liver metastasis | | 2.51 | | 0.79-7.97 | 0.117 |  | 4.06 | 0.78-21.05 | | 0.094 |
| Brain metastasis | | 0.03 | 0.00-38.26 | | 0.355 |  | 0.00 | 0.00-1.34 | | 0.789 |
| PD-L1 status  Positive  (vs. negative)  Unknown  (vs. negative) | | 0.00  0.70 | | 0.00-NR  0.15-3.14 | 0.900  0.978  0.646 |  | 0.00  1.64 | 0.00-1.26  0.24-11.18 | | 0.862  0.857  0.609 |

NR: not reached

**Supplement Table 3.** The summarize of published articles and our study on correlation of inflammation indicators and immunotherapy in patients with advanced non-small cell lung cancer

| **Author** | **Nation** | **NSCLC patients number** | **Treatment regimens** | **dNLR** | **LIPI** |
| --- | --- | --- | --- | --- | --- |
| Suh et al [14] | Australia | 54 (retrospective study) | ICIs monotherapy | Pre- and post- NLR(P＜0.001, PFS、OS） | None |
| Nakaya et al [15] | Japan | 101 (retrospective study) | Nivolumab | Post- NLR(P＜0.001, PFS） | None |
| Mezquita et al [16] | France | 466 (retrospective study+clinical trials) | ICIs monotherapy and durvalumab+ipilimumab | Pre-NLR (P=0.03, PFS) | Pre-LIPI (P＜0.001, OS) |
| Li et al [17] | America | 509 (retrospective study) | ICIs monotherapy and Nivolumab+ipilimumab | pre- and post- NLR (P＜0.001, OS) | None |
| Sorich et al [18] | Australia | 1489  (clinical trials) | Atezolizumab | None | Pre-LIPI (P＜0.001, PFS、 OS) |
| Diem et al [21] | Switzerland | 52 (retrospective study) | Nivolumab | Pre-NLR (P＜0.001, OS） | None |
| Zer et al [22] | Australia | 88 (retrospective study) | ICIs monotherapy | Pre-NLR (P=0.019, OS) | None |
| Hasegawa et al [23] | Japan | 51 (retrospective study) | Pembrolizumab | Pre-NLR (P=0.004, PFS、OS） | None |
| Meyers et al [24] | Canada | 302 (retrospective study) | ICIs monotherapy and Nivolumab+ipilimumab | None | Pre-LIPI( P＜0.001, PFS、 OS) |
| Kazandjian et al [25] | America | 1368  (clinical trials) | ICIs | None | Pre-LIPI (P＜0.001, OS） |
| Our study | China | 314 (retrospective study) | ICIs monotherapy and ICIs with chemotherapy | Pre- and Post-NLR（PFS）  P＜0.05 in ICIs monotherapy  P＞0.05 in ICIs with chemotherapy | Pre- and Post- LIPI  （P＜0.05 in ICIs monotherapy  P＞0.05 in ICIs with chemotherapy, PFS、OS） |
